# The coming era of artificial intelligence in biological data science

**DOI:** 10.1186/s12859-019-3225-3

**Published:** 2019-12-30

**Authors:** Henry Han, Wenbin Liu

**Affiliations:** 1 Department of Computer and Information Science, Fordham University, Lincoln Center, 113 W. 60th Street, New York, NY 10023 USA; 2 Institute of Computational Science and Technology, Guangzhou University, College city 240, Guangzhou, 510006 China

The biological data science is characterized by a massive amount of data from heterogeneous sources. How to decipher complex relationships among heterogeneous datasets remains an urgent challenge. Although traditional model-driven methods still play an important role in analyzing all kinds of data, it lacks capabilities to exploit the huge amount of available data or even big data to discover knowledge, predict data behaviors, and decipher complex relationships among data. Therefore, data-driven becomes the theme of biological data science for its capabilities in listening to data, interacting with data, and extracting knowledge from data.

Modern artificial intelligence will dominate biological data science for its unpreceded learning capabilities to process complex data. Compared to traditional AI techniques (e.g. automated reasoning), machine learning and deep learning are the core to enable machines with intelligence. A deep learning machine has much more complicate learning topologies, which may change dynamically for the sake of learning, besides at least the same complicate-level learning mechanism as traditional machine learning models such as support vector machines.

Deep learning is good at discovering latent complex relationships among data and handling big data well. More importantly, deep learning merges feature extraction and prediction (e.g. classification) in a single learning procedure and makes feature extraction more adaptive and compatible with prediction. The scRNA-seq data, SNP, interactome or even clinical data usually need very different but complicate feature extraction procedures before entering downstream learning. Deep learning prepares itself for a good candidate to process those data and starts to make good progress in handling next generation sequencing data.

Artificial intelligence is expected to dominate biological data science in the near future with the maturity of AI itself. Most state-of-the-art AI techniques are originated from computer vision, image recognition, or natural language processing. It is not easy to migrate the existing AI techniques to the biological data science field though some efforts are being made. The special characteristics of enormous data generated in biological data science calls for building their own AI theory, methods, and systems. To some degree, the maturity of AI in biological data science will indicate the realization of precision medicine.

This special issue aims to initialize AI techniques for bioinformatics, clinical, and health data. All papers included in this special issue have developed their own novel AI techniques in problem-solving. They range from a computational framework for disease-specific gene regulatory network detection to graph regularized low-rank representation for multi-cancer sample clustering, graph-Laplacian PCA, and etc. In particular, one paper in this special issue is devoted to effectively detecting the clinic risk factors of portal vein system thrombosis (PVST) for splenectomy and cardia devascularization patients by building an SVM-based prediction system under novel feature extraction. It presents pioneering research work on this topic though results are still not that perfect. However, it can inspire more future work on the rare-explored topic by using more advanced deep learning techniques (e.g. novel few-shot learning) to extract high-level representative hidden features for the sake of clinic risk analysis.

